# Prokineticin 2 Regulates the Electrical Activity of Rat Suprachiasmatic Nuclei Neurons

**DOI:** 10.1371/journal.pone.0020263

**Published:** 2011-06-08

**Authors:** Ping Ren, Huiping Zhang, Fang Qiu, Yu-Qiang Liu, Huaiyu Gu, Diane K. O'Dowd, Qun-Yong Zhou, Wang-Ping Hu

**Affiliations:** 1 Department of Pharmacology, Xianning College, Xianning, Hubei, People's Republic of China; 2 Family Planning Research Institute, Tongji Medical College, Huazhong University of Science and Technology, Wuhan, People's Republic of China; 3 Departments of Anatomy and Neurobiology, Developmental and Cell Biology, University of California Irvine, Irvine, California, United States of America; 4 Department of Pharmacology, University of California Irvine, Irvine, California, United States of America; University of Cincinnatti, United States of America

## Abstract

Neuropeptide signaling plays roles in coordinating cellular activities and maintaining robust oscillations within the mammalian suprachiasmatic nucleus (SCN). Prokineticin2 (PK2) is a signaling molecule from the SCN and involves in the generation of circadian locomotor activity. Prokineticin receptor 2 (PKR2), a receptor for PK2, has been shown to be expressed in the SCN. However, very little is known about the cellular action of PK2 within the SCN. In the present study, we investigated the effect of PK2 on spontaneous firing and miniature inhibitory postsynaptic currents (mIPSCs) using whole cell patch-clamp recording in the SCN slices. PK2 dose-dependently increased spontaneous firing rates in most neurons from the dorsal SCN. PK2 acted postsynaptically to reduce γ-aminobutyric acid (GABA)-ergic function within the SCN, and PK2 reduced the amplitude but not frequency of mIPSCs. Furthermore, PK2 also suppressed exogenous GABA-induced currents. And the inhibitory effect of PK2 required PKC activation in the postsynaptic cells. Our data suggest that PK2 could alter cellular activities within the SCN and may influence behavioral and physiological rhythms.

## Introduction

The mammalian suprachiasmatic nucleus (SCN) is the master pacemaker controlling daily rhythms in physiology and behavior [1]. Circadian rhythms are generated in individual SCN neurons via positive and negative feedback loops involving transcription and translation of so-called clock genes [1], [2]. The SCN is composed of numerous single-cell oscillators that, when synchronized, produce a coordinated circadian output. Neurochemical and electrical signaling between SCN neurons is necessary for these individual cellular clocks to coordinate their activities and maintain robust oscillations [3]–[5]. One prominent feature of neurons in the SCN is the circadian rhythm in spontaneous firing rate which peaks during the light phase in nocturnal animals [5]. The firing rate of SCN neurons is clearly linked to behavioral and physiological rhythms. The activity of the SCN is thought to suppress daytime locomotor activity [6] by both direct innervation [7] and via the actions of humoral transmitting molecules [8]. Recently, many neurochemical signals have been reported to regulate the electrical activity of SCN neurons [9]–[16].

Prokineticin2 (PK2) has been identified as an output molecule of the SCN and exhibits high circadian rhythmic expression in the SCN [17]–[19]. Transcription of PK2 is tightly controlled by components of the core molecular circadian oscillators [17]. PK2 mRNA expression levels are high during the day and low throughout the night in the SCN of mice and rats [17], [20]–[21]. Intracerbroventricular delivery of PK2 at night, when endogenous levels are minimal, suppresses locomotor activity and feeding behavior [17]. PK2-deficient mice exhibited significantly reduced rhythmicity for a variety of physiological and behavioral parameters, including sleep/wake cycle, locomotor activity, feeding, and body temperature [22]–[23]. Prokineticin receptor 2 (PKR2), a receptor for PK2, has been shown to be expressed in most primary target areas of the SCN by mRNA in situ hybridization [20]–[21]. Recently, Zhang et al reported that PK2-expressing neurons from the SCN projected to many known target areas utilizing a bacterial artificial chromosome transgenic mouse [24]. The circadian phenotypes of PKR2-mutant mice are almost identical with that of PK2- deficient mice [25]. The targeted null mutation of PKR2 disrupts circadian coordination of the activity cycle and thermoregulation. Thus, PK2-PKR2 signaling is critical for the maintenance of robust circadian rhythms.

PK2 has been shown to modulate the electrical activity of neurons through the activation of PKR2 in the area postrema, subfornical organ, and paraventricular nucleus of the hypothalamus [26]–[28]. Interestingly, PKR2 mRNA is also expressed in the SCN [17], [21], and PKR2 mRNA-containing neurons are clustered in the dorsomedial region of the SCN [20], suggesting that these receptors may play a crucial role in regulating neuronal activity of the SCN. In the present study, cell-attached recordings revealed that PK2 increased spontaneous firing rate of dorsal SCN neurons, and whole-cell voltage clamp recordings showed that PK2 reduced the amplitude but not frequency of miniature inhibitory postsynaptic currents (mIPSCs) in the SCN slices.

## Results

### PK2 caused an increased spontaneous firing rate in the SCN neurons

**Figure 1:**
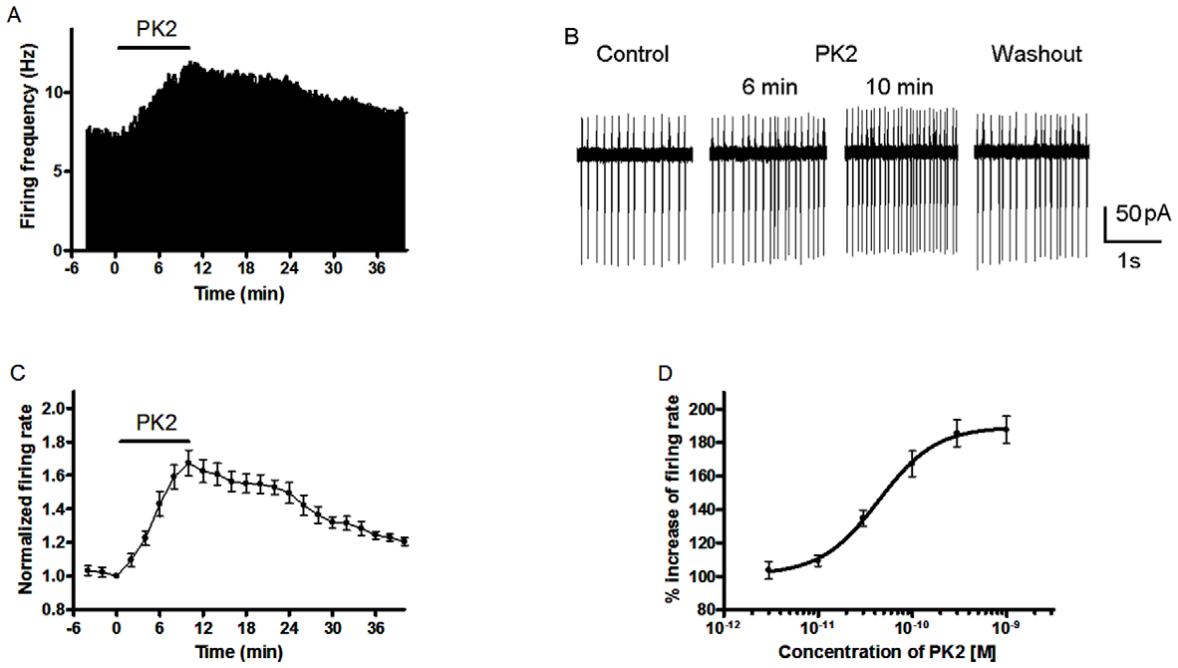
Effect of PK2 on spontaneous firing in suprachiasmatic nuclei (SCN) neurons. (A) Firing rate histogram showing an example of a neuron responding to PK2 (10 min treatment with 0.1 nM) with increased firing. (B) Representative traces of spontaneous activity from the cell with the cell-attached mode in (A). (C) Time course of change in spontaneous firing rate from eight cells exposed to 0.1 nM PK2 for the duration indicated by the length of the line. Data are represented as normalized mean ± SEM. (D) Dose-response curve for the effect of PK2 on spontaneous firing rate. EC_50_ was calculated to be 44.3 pM. Each point represents the normalized mean ± SEM of 6–8 neurons. All firings were recorded in the dorsal division of SCN during daytime (ZT 4–8).

Spontaneous firing in the neurons from the SCN was recorded in the cell-attached configuration of the patch clamp technique. We examined the effects of 10 min application of 0.1 nM PK2 on spontaneous firing rate in eight SCN slices during daytime (ZT4–8). PK2 caused an increased firing rate in eleven of 13 neurons located within the dorsal region of SCN, and two neurons showed no response. In the present study, we established a cut-off value for the effect of PK2, which was at least a 10% the change in the firing rate. Figure 1A and B showed the effect of PK2 on a representative SCN neuron recorded with the cell-attached mode. Application of 0.1 nM PK2 increased the spontaneous firing rate from 7.4 to 13.6 Hz for this particular cell. In 11 neurons responding to PK2 with an increased firing rate, the onset of effect could be seen during the 10 min period of PK2 exposure. Figure 1C illustrated the time-effect relationship of 0.1 nM PK2 on changes in spontaneous firing rate in eight neurons of the dorsal SCN. The potentiation of PK2 on spontaneous firing reached a maximal effect at 10 min after the onset of application, and firing rates increased 67.3±7.7% of the control values of pre-drug. During washout of PK2, spontaneous firing recovered gradually. The effect of PK2 on SCN neurons was concentration dependent with a maximal effect observed at 1 nM and a half-maximal response (EC_50_) at 44.3 pM (Fig. 1D).

### Effects of PK2 on the dorsal and ventral neurons of the SCN

**Figure 2:**
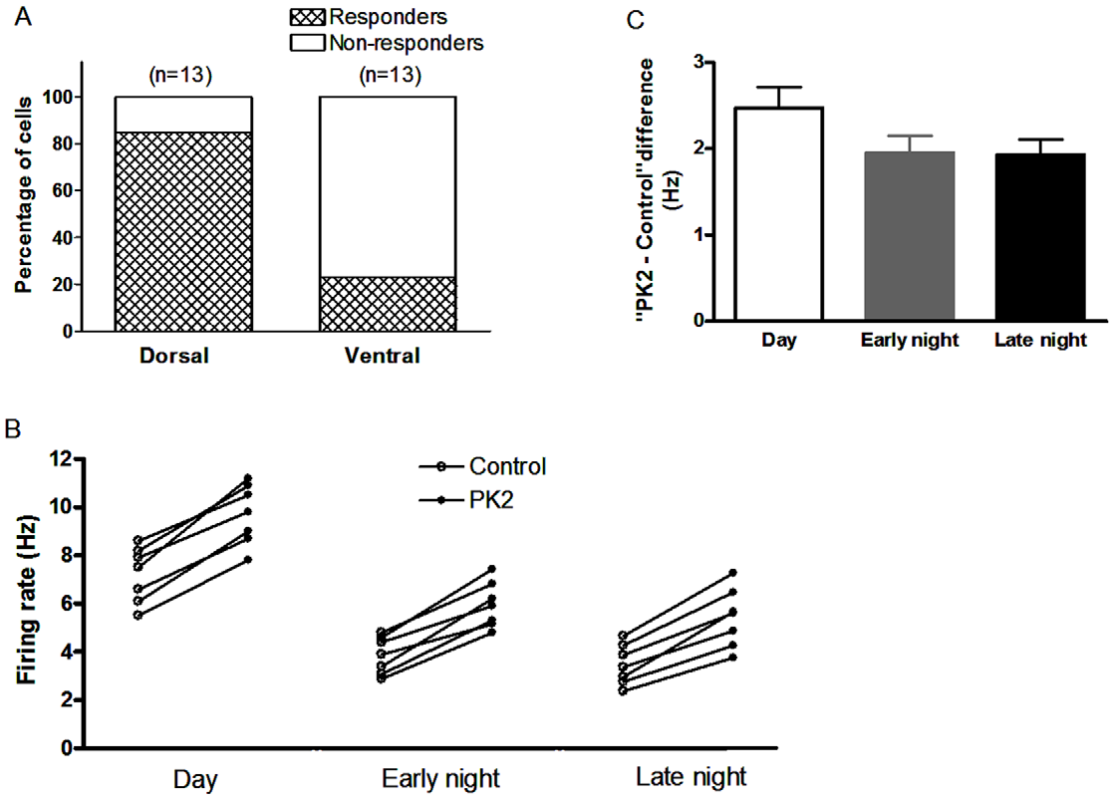
Effects of PK2 on spontaneous firing in different the SCN sub-regions and different the phases. (A) A majority of neurons (11/13) responded to 0.1 nM PK2 with increased firing rate in the dorsal division of SCN, in contrast, only three of 13 cells sensitive to PK2 in the ventral division. Cells that demonstrated at least a 10% the change in the firing rate were considered “responders”. (B) PK2 (30 pM) increased firing rate of SCN neurons during three phases, day (ZT 4–8), early night (ZT 13–15), and late night (ZT 20–22). Each pair represents the firing rate of a neuron before and after PK2 treatment. (C) The histogram showing the “PK2 - Control” differences for firing rate during three phases in (B). There was no difference between the day, early and late night (n = 7, P>0.05, analysis of variance).

The dorsal and ventral regions of the SCN were defined as described previously [29]. There was distinct localization of prokineticin receptor 2 (PKR2) in the subregions of the SCN, and the PKR2-expressed neurons were clustered in the dorsomedial division [20]. Therefore, we compared the effect of PK2 on spontaneous firing rate of the dorsal and ventral SCN. There was not different in the baseline firing frequency between dorsal and ventral SCN neurons in the 13 tested neurons during daytime (ZT4–8). Howerer, the percentages of cells responding to 0.1 nM PK2 were different between the dorsal and ventral neurons of the SCN during daytime ((Fig. 2A)). Of all 13 neurons examined, eleven cells (84.6%, 11/13) responded to PK2 with increased firing rate in the dorsal SCN, whereas only three of 13 cells (23.1%, 3/13) sensitive to PK2 in the ventral division (P<0.05, χ^2^ test). Neurons that demonstrated at least a 10% the change in the firing rate were considered “responders” in the present study. Thus, we performed experiments in the dorsal division of the SCN unless otherwise indicated.

### Effect of PK2 on spontaneous firing rate during different phases in the SCN neurons

The neurons from the SCN have a higher firing rate during the day than at night [5]. And the PK2 expression in the SCN is higher during the day. To see if PK2's effect on the spontaneous firing rate shows day-night variation, we investigated the effect of PK2 during three different ZT phases. During the day (ZT 4–8), Application of PK2 at low concentration (30 pM) increased the firing rate from 7.2±0.4 to 9.7±0.5 Hz (n = 7, P<0.01, paired t-test) (Fig. 2B). Similarly, PK2 treatment increased the firing rate from 3.9±0.3 to 5.9±0.4 Hz (n = 7, P<0.01, paired t-test) during the early night (ZT 13–15) and from 3.5±0.3 to 5.4±0.5 Hz (n = 7, P<0.01, paired t-test) during the late night (ZT 20–22) (Fig. 2B). Nevertheless, there was no temporal difference in the absolute PK2-induced firing rate change. The “PK2 - control” differences for firing rate during three phases were 2.5±0.2, 2.0±0.2 and 1.9±0.2 Hz during the day, the early and late night, respectively (Fig. 2C; P>0.05, analysis of variance).

### PK2 reduced the amplitude but not frequency of mIPSCs in the SCN

**Figure 3:**
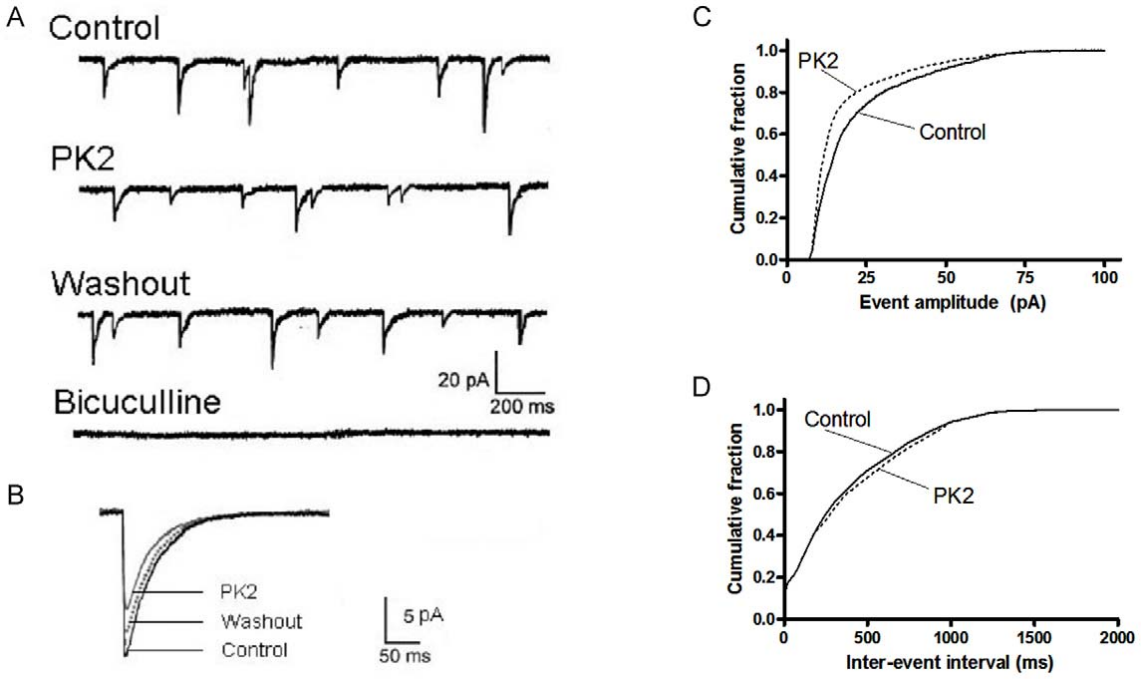
Effect of PK2 on GABA_A_ receptor-mediated miniature inhibitory postsynaptic currents (mIPSCs) recorded from rat SCN neurons. (A) Representative current traces showing mIPSCs obtained under control conditions, during application of 0.1 nM PK2 and washout conditions. The slice was bathed in AP5 (50 µM), DNQX (10 µM), and TTX (1 µM). The holding potential was −70 mV. Bath application of 10^−10^ M PK2 for 10 min reduced the amplitudes of mIPSCs, but did not affect their frequency. During washout of PK2, there was a partial recovery of mIPSC amplitude. The mIPSCs were blocked by 30 µM bicuculline. (B) Average mIPSC waveforms of 300 randomly selected mIPSCs for each condition. (C, D) Cumulative event amplitude and inter-event interval plots for the neuron in A before and after PK2 application. PK2 caused a leftward shift in the amplitude but not interval distribution indicating that PK2 decreased the amplitude of mIPSC without altering the frequency.

To test whether the effects of PK2 on spontaneous firing were associated with changes in synaptic transmission, we performed recordings of mIPSCs from neurons in the dorsal SCN using whole cell voltage-clamp technique during daytime (ZT 4–8). The mIPSCs were pharmacologically isolated by applying a combination of APV (50 µM) and DNQX (10 µM) to block NMDA and AMPA/kainite receptors, respectively, and TTX (1 µM) to block Na^+^ channel-mediated action potentials and the postsynaptic Na^+^ currents from them. All remaining currents were GABA_A_-receptor-mediated currents and could be suppressed by the GABA_A_ receptor antagonist bicuculline (30 µM) (Fig. 3). The mIPSCs recording in this manner appear as inward currents, since the holding potential (−70 mV) was more negative than the chloride equilibrium potential. Bath application of 0.1 nM PK2 for 10 min was found to reduce the amplitudes of mIPSCs, but did not affect their frequency (Fig. 3A and B). During washout of PK2, The mIPSC amplitude recovered partially. The effect of PK2 was widespread in the dorsal SCN. In ten of the neurons examined, eight cells responded to PK2 exposure. The average amplitudes of mIPSC for these eight cells in the presence of PK2 decreased to 65.7±7.2% of the control period (P<0.01, paired t-test), and the average frequency was 107.4±13.4% of the control period (P>0.05, paired t-test). The remaining two cells were not sensitive to PK2 and changes in the average amplitude and frequency were within 5%. If we consider all ten cells tested as a single population, then PK2 caused a 28.1±3.4% reduction in the average amplitudes of mIPSC (P<0.01, paired t-test). Figure 3C showed the average mIPSC amplitude distribution of the neuron tested for the control and PK2 condition in a cumulative fraction plot. PK2 caused a shift of the curve towards smaller amplitudes. In contrast, PK2 did not affect the frequency of mIPSCs as shown in a cumulative fraction plot for the averaged interevent interval for each condition (Fig. 3D). In addition, PK2 did not alter mIPSC kinetics and no difference was found in the rise time constants (2.1±0.2 ms with PK2 pretreatment vs. 2.0±0.2 ms without PK2 pretreatment) and decay time constants (20.6±1.9 ms with PK2 pretreatment vs. 22.4±2.3 ms without PK2 pretreatment) of mIPSC after treatment of PK2.

### PK2 inhibited exogenous GABA-induced currents in SCN neurons

**Figure 4:**
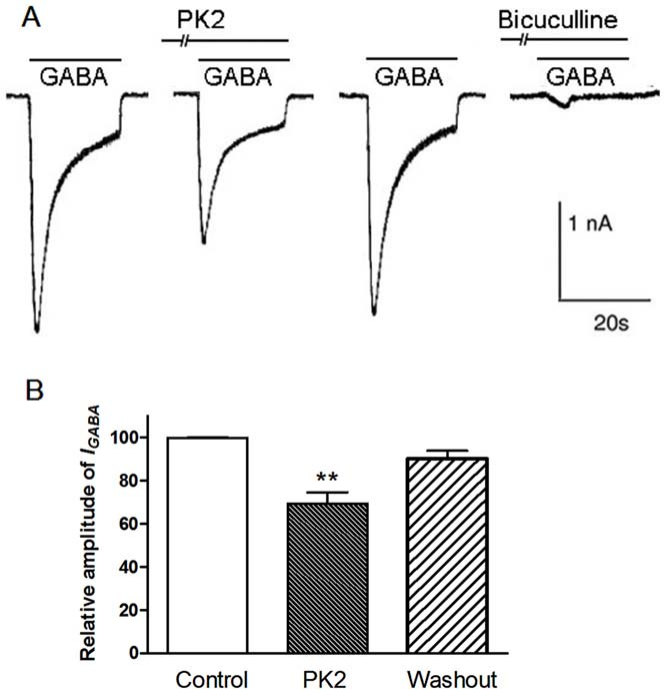
Inhibition effect of PK2 on exogenous GABA-induced currents in SCN neurons. (A) The inward currents were elicited by 0.1 mM GABA in a SCN neuron voltage clamped at −70 mV. PK2 exerted an inhibitory effect on the GABA-activated current. PK2 (0.1 nM) was pre-applied to external solution for 10 min. The GABA-activated current could be blocked by 30 µM bicuculline. (B) A summary bar graph showing PK2 had significant effect on the responses to exogenous application of GABA (*P<0.05, n = 7).

We previously reported that PK2 depresses GABA-induced currents in the primary sensory neurons [30]. To further investigate the site of modulatory action of PK2 on GABA_A_ receptor signaling in SCN neurons, we next examined the effect of PK2 on exogenous GABA-induced currents. Exogenous application of GABA (0.1 mM) produced an inward current at holding potential −70 mV in SCN neurons during the day. This GABA-evoked current could be blocked by GABA_A_ receptor antagonist bicuculline (30 µM). Pre-treatment with 0.1 nM PK2 for 10 min, peak GABA-induced currents were significantly different of those observed before application of PK2. The amplitude of GABA-activated currents decreased to 66.9±5.2% of the control in seven neurons tested (Fig. 4). Thus PK2 may act at the postsynaptic GABA_A_ receptor.

### Depression of GABA_A_ receptor-mediated current by PK2 required postsynaptic kinase C

PKR2, a receptor of PK2, belongs to Gq protein-coupled receptors and mainly expresses in dorsomedial SCN neurons [20]. Next we analyzed intracellular signal route concerned in the suppression of GABA_A_ receptor-mediated current by PK2. GF109203 X (2 µM), a selective PKC inhibitor, was applied internally to SCN neurons through recording patch pipettes. PK2 (0.1 nM) did not depress mIPSCs in seven neurons treated with GF109203X, and the change in average amplitude of mIPSCs was 5.7±4.1%. In contrast, PK2 (0.1 nM) caused a 31.1±3.8% (n = 7) depression of mIPSCs when the pipette filled with normal internal solution (P<0.01, t-test). The baseline frequency and average amplitude of mIPSCs were not different in the presence and absence of GF109203X, and the changes induced by GF109203X were within 3.0%. Intracellular application of GF109203X did not alter mIPSC kinetics. Similarly, the effect of PK2 on exogenous GABA-evoked currents was also prevented by the internal treatment of GF109203 X. PK2 caused only a 9.7±4.1% (n = 7) depressive effect on exogenous GABA-evoked currents in neurons treated with GF109203 X, while PK2 depressed GABA-evoked currents by 33.1±5.2% (n = 7) when the pipette filled with internal solution (P<0.01, t-test). Thus, PKC may play a key role in the regulation of GABA_A_ receptor in SCN neurons.

## Discussion

These data demonstrated the effects of PK2 on electrical activity of rat SCN neurons. Firstly, PK2 dose-dependently increased the spontaneous firing rate of SCN neurons, and the proportion of cells responding to PK2 was greater in the dorsal region than the ventral region. Secondly, PK2 inhibited GABA_A_-receptor signaling within the SCN. The site of PK2 action was postsynaptic, since PK2 reduced the amplitude but not frequency of mIPSCs. Furthermore, PK2 also suppressed exogenous GABA-induced currents. Finally, the inhibitory effect of PK2 required PKC activation in the postsynaptic cells.

It is reported that PK2 produces an excitatory role on the other neurons from subfornical organ and paraventricular nucleus of the hypothalamus through depolarizing membrane potentials and modulating Na^+^ channels [27]–[28]. As the PKR2 is highly expressed in the SCN, the effect of PK2 on SCN neurons may be mediated by PKR2. The SCN consists of two morphologically and functionally distinct regions, the ventrolateral (or core) and dorsomedial (or shell) SCN [31]–[32]. These two regions have distinct afferent inputs. The ventrolateral region receives direct input from the retina through the retinohypothalamic tract (RHT) and is thus photo-responsive [31]. In contrast, the dorsomedial region, lacking the projection from outside of the SCN, is modulated by entraining input from the ventrolateral region [33]. Interestingly, PKR2 mRNA-containing neurons were clustered in the dorsomedial region of the SCN [20]. In the present study, the majority of neurons responding to PK2 located in the dorsal SCN, suggesting that the effect of PK2 was mainly restricted to dorsal region, consistent with the localization of PKR2 mRNA in the SCN. PK2, as an output molecule, is highly expressed in the SCN and its mRNA level displays a robust circadian oscillation with a peak occurring during the day [17]. PK2 mRNA-positive neurons are scattered in both the dorsomedial and ventrolateral SCN [20]. Thus, PK2 neurons may intrinsically project to the PKR2-expressing neurons in the dorsal SCN. Rhythmicity of neuronal activity in the SCN relies on both intrinsic currents that generate the day-night difference in activity, as well as intercellular communication to synchronize the rhythms of individual neurons. Our data indicated that PK2-PKR2 signaling may play an important modulatory role in regulating cell-to-cell communication within the SCN. Spontaneous firings in the SCN show a diurnal pattern. Neurons are more active during the day, and activity is suppressed at night [6]–[7]. Recently, Belle et al reported that per1 cells were completely silent during the afternoon and early night and showed different daily variation in firing frequency compared to non-per1 cells in the SCN [34]. In the present study, the firing patterns of neurons recorded were similar to the patterns of non-per1 cells, with high rates during the day and lower rates at night. In the SCN, the enhanced effect of PK2 on spontaneous firing was consistent with higher firing rates and PK2 mRNA expression levels during the day.

It is widely accepted that most SCN neurons are GABA-ergic and this transmitter plays an important role in coordinating interneuronal communication both within and between dorsal and ventral SCN subcompartments [35]–[36]. SCN neurons are under tonic GABAergic control and receive a high frequency of GABA_A_-mediated postsynaptic currents [37]–[40] that at least partly originate within the SCN itself [41]. Most studies examining the effects of GABA on rodent SCN neurons demonstrate that its actions are inhibitory and are predominantly mediated via GABA_A_ receptors [5], [42]–[43]. We previously reported that PK2 depresses GABA-induced currents in the primary sensory neurons [30]. In agreement with the enhanced effect of PK2 on firing rate, we observed that PK2 reduced the amplitude of mIPSCs through suppressive GABA_A_ receptor signaling in the SCN. It is therefore likely that PK2 gives rise to changes in firing rate by influencing synaptic strength and suppressing GABAergic inhibition within the SCN. In the present study, we confirmed that PK2 depressed postsynaptic sensitivity of GABA_A_ receptors, rather than changing the release of GABA by demonstrating that it reduced amplitude but not the frequency of mIPSCs. The postsynaptic depressant effect of PK2 on GABAergic synapses was further supported by the observation that PK2 decreased postsynaptic responses to exogenously applied GABA.

The inhibition of PK2 on the GABA_A_ receptors-medicated currents appeared to involve PKC signal pathway, since this effect was completely prevented by abolishing postsynaptic PKC activation with the selective PKC inhibitor GF109203 X. PKR2 is the most likely target to mediate PK2 inhibitory effect on GABA currents, since PKR2 mRNA is highly expressed in the dorsomedial region of the SCN [20]. PKR2 belongs to the G-protein receptor and is couple to Gq protein to mediate the intracellular calcium mobilization and the activation of PKC [44]–[46]. There are already good evidences that GABA currents are negatively regulated by PKC [47]–[49]. Henneberger et al [50] reported that BDNF reduced mIPSC amplitudes in a PKC dependent manner in superior colliculus slices. The observation that PK2 decreased GABAergic mIPSCs may due to PKC-mediated internalization, rather than phosphorylation of GABA_A_ receptors, since no changes were found in the kinetics of mIPSCs. It is shown that the cell surface stability of GABA_A_ receptors depends on PKC activity [51]–[53] and GABA_A_ receptor numbers in the plasma membrane were reduced on PKC activation [54].

In summary, we showed that PK2 increased the spontaneous firing rate and inhibited GABA_A_-receptor signaling within the SCN. Such effect on SCN neuronal activity may influence behavioral and physiological rhythms.

## Materials and Methods

### Animals

All experiments were approved by the Institutional Animal Care and Use Committee of Xianning College (approval no. 1016) and were carried out in strict accordance with the National Institutes of Health Guide for the Care and Use of Laboratory Animals. Male Sprague-Dawley rats (17–26 day) were housed under a 12 ∶ 12 light ∶ dark cycle (light on 7:00 am–7:00 pm) at an ambient temperature of 22±1°C. Food and water were available *ad libitum*. Zeitgeber time (ZT) 0 was defined as lights-on and ZT 12 as lights-off. Animals were maintained under these conditions for >10 days prior to experimental protocols.

### SCN slice preparation

Slices were prepared during the day (ZT2), early night (ZT11) or late night (ZT18), and maintained using methods similar to those described earlier [11], [29]. Rats were decapitated under deep isofluorane anesthesia. Brains were then rapidly dissected and immersed in ice-cold artificial cerebral spinal fluid (ACSF) pre-bubbled with 95% O_2_-5% CO_2_. The ACSF contained 125 mM NaCl, 3.5 mM KCl, 2 mM CaCl_2_, 1.5 mM MgCl_2_, 26 mM NaHCO_3_, 1.2 mM NaH_2_PO_4_, and 10 mM glucose. Coronal slices (200–300 µm) containing the SCN and the optic chiasm were cut using a vibroslicer (Campden Instruments, Leicester, UK). Slices were then transferred to holding chamber and incubated for >1 h in oxygenated ACSF at 36°C before the start of electrophysiological experiments. The ventral or dorsal region of the SCN is defined as the upper or lower one third of the SCN, by drawing two imaginary lines parallel to the optic chiasm and dividing the SCN into three approximately equal-size divisions [11], [29].

### Electrophysiological recordings and data analysis

All experiments were performed during the daytime (ZT 4–8), unless stated as early night (ZT13–15) or late night (ZT 20–22). Slices were continuously perfused (∼1.5 ml/min) with oxygenated ACSF and maintained at a temperature of 36°C. Electrodes (resistance 4–6 MΩ) were pulled from borosilicate (1.5 mm OD) on a horizontal puller (P-97; Sutter Instrument, CA, USA). The spontaneous firing was recorded in the cell-attached voltage-clamp mode without membrane breakthrough. The patch electrode solution was the same as the bath solution. The spike counts, in 6-sepochs, always began only after stable recordings were made. At least 1 or 2 min of spontaneous firing rate were counted before the application of drugs. Whole-cell recordings were made in voltage-clamp mode at a holding potential of −70 mV for analysis of postsynaptic currents. Pipettes were filled with a solution containing (in mM) 130 CsCl, 10 HEPES, 1 NaCl, 1 CaCl_2_, 1 MgCl_2_, 5 EGTA, 2 ATP, 0.2 GTP, and 5 QX314, and pH adjusted to 7.2–7.3 with CsOH. Whole cell patch-clamp recordings were made using infrared illumination with differential interference contrast optics (IR-DIC) video microscopy. Seal resistances were typically 1–4 GΩ, and series resistances were <20 MΩ. Whole cell currents were amplified with an Axopatch-Multiclamp700A amplifier (Axon Instruments) and digitized with a Digidata 1440A (Axon Instruments). Records were collected and stored onto a PC using the pCLAMP 8.0 software (Axon Instruments). Miniature IPSCs were isolated by using the glutamate AMPA/kainite receptor antagonist, 5,7-dinitroquinoxaline-2,3-dione (DNQX; 10 µM), the N-methyl-D-aspartate (NMDA) receptor antagonist DL-2-amino-5- phosphonopentanoicacid (APV; 50 µM), and tetrodotoxin (TTX; 1 µM). All parameters of postsynaptic currents were analyzed off-line with Mini-Analysis software (Synaptosoft). All values are given as mean ± SEM, and statistical analysis was performed using the Student's *t*-test and χ^2^ test.

### Drugs

PK2 was obtained from our lab. DNQX, APV, TTX, GABA, and GF109203 X were purchased from RBI/Sigma. All drugs were dissolved daily just before use and applied to the cells by bath perfusion of slices with ACSF containing the concentration of the drug, but GF109203X that needed to be applied intracellularly was dissolved in the internal solution.
